# L’intérêt du trauma damage control orthopédique dans les polytraumatismes : un cas de disjonction du bassin associée à une luxation de la hanche avec atteinte vasculaire

**DOI:** 10.11604/pamj.2017.27.122.8699

**Published:** 2017-06-15

**Authors:** Tarik Madani, El Mehdi El Alouani, Younes Mhammdi, Mohammed Kharmaz, Mohamed El Ouadghiri, Abdou Lahlou, Lamrani Moulay Omar, Ahmed El Bardouni, Mustapha Mahfoud, Mohamed Saleh Berrada

**Affiliations:** 1 Service de Chirurgie Orthopédique et Traumatologique du Centre Hospitalier Universitaire de Rabat, Maroc

**Keywords:** DCO, polytraumatisme, bassin, TDCO, polytrauma, pelvis

## Abstract

La connaissance de la physiopathologie du traumatisé grave et les conséquences hémodynamiques et inflammatoires de la prise en charge chirurgicale initiale a amené de nombreux chirurgiens à modifier leur approche du traitement des polytraumatisé graves avec lésions du bassin ou des membres en intégrant les principes d'un traitement séquentiel ou Trauma Damage Control Orthopédique (TDCO).Nous rapportons le cas d'une patiente victime d'un accident de la voie publique, admise dans un tableau d'état de choc avec un disjonction du bassin associée à une luxation de la hanche compliquée d'une lésion vasculaire du même membre. Nous avons agit selon les concepts du TDCO en privilégiant une fixation externe du bassin après réduction de la luxation. La rapidité de notre conduite a permis une revascularisation précoce du membre tout en évitant les complications hémodynamiques et inflammatoires de la chirurgie à ciel ouvert.

## Abstract (en)

The knowledge of the pathophysiology of patients with severe trauma and the hemodynamic and inflammatory consequences of initial surgical management has led many surgeons to change their approach to the treatment of patients with severe polytraumas associated with lesions of the pelvis or of limbs by integrating the principles of sequential treatment or Trauma Damage Control Orthopaedic (TDCO). We report the case of a patient involved in a public road accident, admitted to hospital in a state of shock with pelvic disjunction and hip dislocation complicated by vascular injury in the same limb. Our approach was based on TDCO concepts by favoring external fixation of the pelvis after hip dislocation reduction. The timeliness of our apprach allowed early limb revascularization while avoiding the hemodynamic and inflammatory complications of open surgery.

## Introduction

Le « damage control » chirurgical a été initialement développé pour faire face aux traumatismes abdominaux avec hémorragie massive. Le concept s'est ensuite étendu à la chirurgie orthopédique pour la prise en charge des polytraumatisés instables ayant des fractures des os longs et du bassin. Le fixateur externe utilisé dans le cadre d'une procédure de « damage control » orthopédique (DCO) est un appareillage temporaire répondant à des exigences spécifiques. Nous rapportons l'observation d'un cas d'une prise en charge réussie d'un polytraumatisme associé à une atteinte vasculaire selon les concepts de (DCO).

## Patient et observation

**Figure 1:**
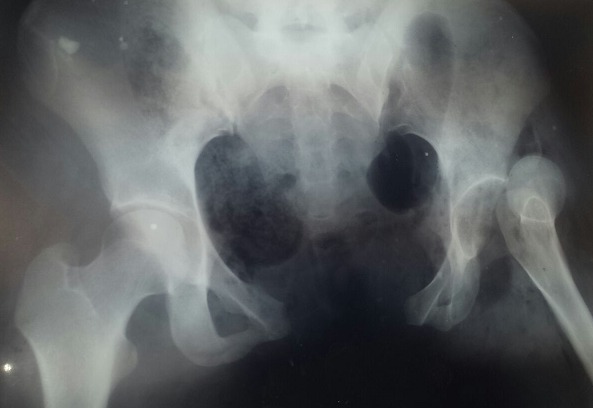
Disjonction du bassin associée à une luxation de la hanche gauche

**Figure 2:**
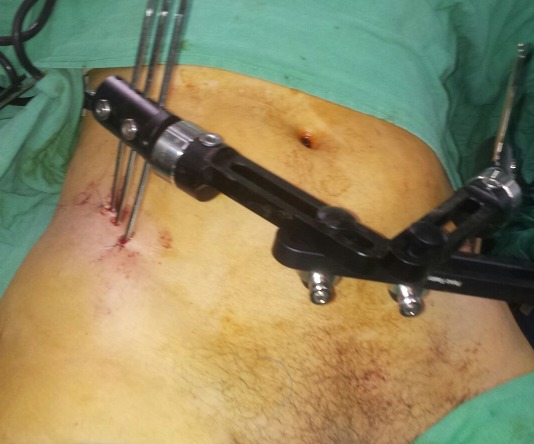
Fixation du bassin par un fixateur externe type orthofix

**Figure 3:**
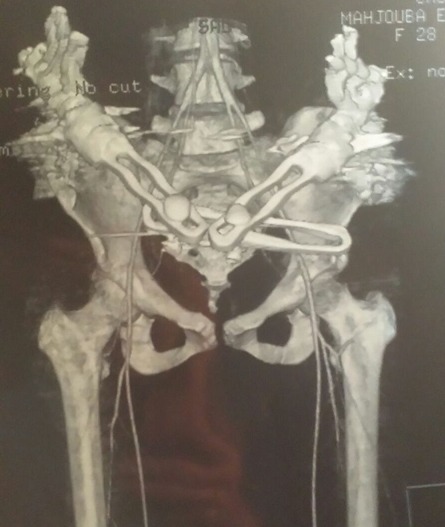
Angioscanner montrant la réduction de la disjonction du bassin

Nous rapportons le cas d'une patiente de 32 ans, admise aux urgences du centre hospitalier universitaire de Rabat pour un polytraumatisme suite à un accident de la voie publique, la patiente s'est éjectée d'une motocyclette après avoir heurté une voiture. La patiente a été admise en état de choc hémorragique (TA 7O/40 mmhg) avec un saignement actif provenant du vagin et une plaie au niveau de la face postero-interne de genou du même membre. L'examen clinique trouve une instabilité du bassin avec un membre inférieur gauche raccourci en extension et rotation interne avec abolition du pouls poplité, le toucher vaginal a révélé une déchirure des parois vaginales avec un saignement actif. Une radiographie du bassin a objectivé une disjonction du bassin supérieure à 3 cm associée à une luxation supéro-externe de la hanche (Figure 1), une échographie abdominale faite à la salle de déchoquage a montré un épanchement de moyenne abondance au niveau du péritoine. La patiente a été admise au bloc opératoire en urgence après stabilisation des fonctions vitales pour stabilisation des lésions du bassin selon les principes du Trauma Damage Control Orthopédique (TDCO). Au bloc, patiente installée en décubitus dorsal nous avons réduit la luxation de la hanche gauche par une traction sur le genou fléchi en maintenant la hanche en flexion, en adduction, la réintégration de la tête dans le cotyle a été réussie avec une bonne stabilité (Figure 2, Figure 3). Dans un deuxième temps, après drapage et badigeonnage nous avons réduit la disjonction pubienne par une pression exercée sur les deux crêtes iliaques de part et d'autre puis stabilisation par un fixateur externe type Orthofix en utilisant trois fiches insérée dans chaque crête iliaque. A l'exploration de la plaie du genou, nous avons diagnostiqué une rupture de l'artère poplitée avec atteinte de l'aileron rotulien interne. Une revascularisation a été réalisée avec succès par un potage en utilisant la veine saphène interne comme greffon. Apres sutures des parois vaginales, la patiente est sortie de l'état de choc hémorragique puis transférée dans un service de réanimation. Pour notre patiente, vu le pronostic vital et aussi fonctionnel du membre, le Trauma Damage Control Orthopédique a permis d'écourter le temps opératoire diminuant ainsi l'importance du « choc chirurgical » et permettant une prise en charge réanimatoire efficace.

## Discussion

La connaissance de la physiopathologie du traumatisé grave et les conséquences hémodynamiques et inflammatoires de la prise en charge chirurgicale initiale a amené de nombreux chirurgiens à modifier leur approche du traitement des polytraumatisé graves avec lésions du bassin ou des membres en intégrant les principes d'un traitement séquentiel ou Trauma Damage Control Orthopédique (TDCO) [1]. En 1983, Stone [2] a décrit la technique de la laparotomie écourtée en de privilégiant des mesures temporaires destinées à contrôler l'hémorragie pour pouvoir maîtriser la coagulopathie, les désordres métaboliques, hémodynamiques et respiratoires en réanimation. Un geste définitif d'hémostase n'était envisagé qu'après restauration d'une homéostasie satisfaisante. Il s'agit d'un traitement chirurgical de sauvetage, issue de l'expérience des conflits militaires, basé sur des gestes simples et rapides et permettant un contrôle rapide de l'homéostasie en réanimation. De nombreux auteurs ont décrit par la suite les effets bénéfiques de ce concept appelé maintenant « damage control ». Les indications sont maintenant bien définies pour les patients polytraumatisés [3]. Elles sont d'ordre général, c'est le cas du polytraumatisé avec lésions vitales associées, ou locorégional dans le cas d'un traumatisme isolé d'un membre grave et d'ordre contextuel en cas de limitation en moyens [4, 5].

**Polytraumatismes avec lésions vitales :** La fixation définitive précoce n'est pas recommandée en cas d'instabilité hémodynamique liée à un traumatisme thoracique, abdominal, cérébral ou du bassin. L'objectif est d'éviter une aggravation de lésions vitales par un geste chirurgical qui dure et agressif (« the second hit ») [6]. Il faut alors recourir au TDCO qui se limite au contrôle de l'hémorragie, au traitement des parties molles et à la stabilisation temporaire des fractures par fixateur externe. Les fractures ou disjonctions de l'anneau pelvien compliquées d'un choc hémorragique indiquent le TDCO ( le cas notre patiente). Le saignement peut être lié à la fracture, à des lésions des artères iliaques ou des plexi veineux. Le bassin doit être refermé au plus vite pour arrêter le saignement pour limiter l'expansion de l'hématome pelvien par effet de tamponnement. En TDCO la stabilisation indispensable est réalisée par les méthodes les moins invasives : moyens orthopédiques (minerve pour le rachis, attelles et plâtre au membre supérieur), fixateur externe temporaire et traction pour les fractures des os longs, ceinture et clamp pelvien pour la stabilisation du bassin [7, 8]. Une fois le patient stabilisé une fixation interne définitive est indiquée, après régression de la réaction inflammatoire et de l'œdème tissulaire. Selon certains auteurs la conversion doit être effectuée au minimum quatre jours après la chirurgie initiale [9].

**Traumatismes sévères isolés.**

**Lésions des membres avec ischémie:** Une fracture associée à une lésion artérielle nécessite une stabilisation rapide des segments osseux par fixateur externe selon une procédure de TDCO pour permettre une réparation vasculaire par la suite. L'emploi d'un fixateur articulé permet une réduction rapide et approximative de la fracture par ostéotaxis, permettant la réalisation du geste vasculaire. Pour les fracture des os longs avec lésion de l'artérielle, il faut utiliser 2 x 2 fiches (ou trois) reliées par une barre de gros diamètre, deux barres en hémi-cadre ou par le corps d'un fixateur monobloc. Les fiches doivent être implantées en per-cutané.

**Le polyfracturé ou les lésions étagées avec fracture du fémur:** Une stabilisation temporaire rapide par fixation externe permet de compléter à tête reposée le bilan et de prendre une décision collégiale pour une meilleure prise en charge définitive et qui peut imposer un matériel spécifique non disponible sur le champ.

**Les fractures impossibles à aborder en urgence pour des raisons de souffrance cutanée:** L'association de lésions osseuses complexes et d'une atteinte des parties molles impose parfois un DCO local, qualifié de « limb damage control orthopaedics » par Roberts et al. [7]. Il s'agit des fractures à haute énergie dans une région ou la couverture par les parties molles est réduite au revêtement cutané, avec deux localisations principales l'extrémité proximale et distale de la jambe, la stabilisation par un fixateur externe permet une surveillance de la peau et des tissus mous et permet de reporter la fixation définitive à plus tard permettant secondairement une ostéosynthèse interne souvent indispensable à l'obtention d'une réduction anatomique.

**Indications par défaut de moyens:** En cas de limitation en moyens humains ou techniques le TDCO est indiqué, c'est le cas d'un blessé qui nécessite un transfert dans une structure plus spécialisée, l'efficacité des évacuations sanitaires aériennes permet actuellement un emploi large des procédures de TDC, en particulier pour la prise en charge des traumatismes des membres [4, 5]. Ce manque de moyen peut être liée à un plateau technique limité (en terme d'infrastructure, de matériel d'ostéosynthèse, de possibilité de réanimation, de compétences chirurgicales), à un environnement d'insécurité ou un afflux massifs de blessés. Le TDCO trouve une application emblématique dans la prise en charge des traumatismes des membres chez les blessés de guerre [5, 10].

## Conclusion

L'objectifs du DCO dans notre observation était double : sauver la vie du polytraumatisé en état de choc en limitant l'agression chirurgicale à la phase aigue et de sauver un membre en ischémie en permettant la réparation vasculaire après réduction de la luxation. L'emploi de la fixation externe dans ce contexte obéit à des règles spécifiques, et repose sur des montages rapides à mettre en œuvre. Nous concluons qu'appréhender le traumatisme, le traumatisé dans sa globalité et le contexte environnant est la clef de l'indication entre un traitement définitif et une procédure de TDCO qui n'est finalement que la formalisation tactique et la conceptualisation de pratiques conservatoires utilisées basées sur le bon sens chirurgical.

## Conflits d’intérêts

Les auteurs ne déclarent aucun conflit d'intérêts
